# Relationship of Fungal Vaginitis Therapy to Prior Antibiotic Exposure

**DOI:** 10.1080/10647440300025514

**Published:** 2003

**Authors:** Douglas D. Glover, Bryan Larsen

**Affiliations:** 1 Professor of Obstetrics and Gynecology West Virginia University Medical Center Morgantown WV 26506-9186 USA; 2 Dean for University Research Des Moines University-Osteopathic Medical Center 3200 Grand Avenue Des Moines IA USA

## Abstract

*Objective:* To address the putative association of antibiotic use and subsequent yeast vaginitis in a population of non-pregnant women.

*Methods:* Three hundred and sixteen women who received medical care in rural family medicine clinics enrolled in this study. Participants were pre-menopausal and non-pregnant and were followed until they used a course of antifungal therapy for vaginitis, became pregnant or moved from the catchment area. At entry subjects were free of vaginitis symptoms and had taken no antibiotics for 30 days. Patients were followed by repeated review of clinic records, hospital records and telephone or personal interviews. Data collection included documentation of episodes of antifungal treatment for vulvovaginal candidiasis and confirmed antibiotic treatment or credible history of antibiotic use prior to the use of antifungal therapy. Physician-reported uses of antibiotic and antifungal as well as patient-reported uses of these were recorded.

*Results:* There were four reported cases of antifungal therapy following within a month of antibiotic use, in contrast to 484 antibiotic uses not followed by antifungal use. If time of observation was extended to 6 months from antibiotic use, there were 13 uses of antifungal therapy after antibiotics and 475 uses of antibiotics not followed by antifungal therapy.

*Conclusion:* Our results cast doubt on the association of antibiotics as a putative cause of yeast vulvovaginitis.


*Infect Dis Obstet Gynecol 2003;11:157–160*

### Relationship of fungal vaginitis therapy to prior antibiotic exposure

**Douglas D. Glover[1] and Bryan Larsen[2]**

[1]*Professor of Obstetrics and Gynecology, West Virginia University Medical Center, Morgantown, WV*
[2]*Dean for University Research, Des Moines University-Osteopathic Medical Center, 3200 Grand Avenue, Des Moines, IA*

***Objective:*** To address the putative association of antibiotic use and subsequent yeast vaginitis in a population of non-pregnant women.
***Methods:*** Three hundred and sixteen women who received medical care in rural family medicine clinics enrolled in this study. Participants were pre-menopausal and non-pregnant and were followed until they used a course of antifungal therapy for vaginitis, became pregnant or moved from the catchment area. At entry subjects were free of vaginitis symptoms and had taken no antibiotics for 30 days.

Patients were followed by repeated review of clinic records, hospital records and telephone or personal interviews. Data collection included documentation of episodes of antifungal treatment for vulvovaginal candidiasis and confirmed antibiotic treatment or credible history of antibiotic use prior to the use of antifungal therapy. Physician-reported uses of antibiotic and antifungal as well as patient-reported uses of these were recorded.
***Results:*** There were four reported cases of antifungal therapy following within a month of antibiotic use, in contrast to 484 antibiotic uses not followed by antifungal use. If time of observation was extended to 6 months from antibiotic use, there were 13 uses of antifungal therapy after antibiotics and 475 uses of antibiotics not followed by antifungal therapy.
***Conclusion:*** Our results cast doubt on the association of antibiotics as a putative cause of yeast vulvovaginitis.

Keywords: FUNGAL VAGINITIS; YEAST VAGINITIS; VULVOVAGINAL CANDIDIASIS; ANTIBIOTIC THERAPY; ANTIFUNGAL THERAPY; ANTIBACTERIAL DRUGS

A commonly held opinion among gynecologists is that antibiotic therapy predisposes women to development of symptomatic vulvovaginal yeast infection. A previous examination of this issue among a cohort of pregnant women indicated that while vaginal colonization is a risk factor for subsequent development of symptomatic yeast vaginitis, antibiotic therapy – even intensive therapy – is not associated with an increased risk of developing symptoms[1]. It might be argued this lack of association was obscured by an increased risk of yeast colonization and symptomatic infection associated with pregnancy. Consequently, we undertook an observational study to address the putative association of antibiotic use and yeast vaginitis in a population of non-pregnant women.

### METHODS

The West Virginia University Institutional Review Board for Protection of Human Research

Correspondence to: Douglas D. Glover, MD, Consortium on Reproductive and Developmental Health of the Robert C. Byrd Health Sciences Center, West Virginia University, Morgantown, WV 26506-9186, USA. Email: dglover2@wvu.edu

© 2003 The Parthenon Publishing Group 

Subjects approved this prospective observational study of the association of antibiotic treatment history and subsequent treatment for vulvovaginal candidiasis. Participants for this study were drawn from women who received their medical care at four Family Medicine clinics in Northern West Virginia and Southwest Pennsylvania. One hundred and eighty-four women followed in this study were identified from participants of a prior study of antibiotic use and vulvovaginitis in pregnancy[1] and were 6 weeks postpartum at the time of entry into the study. An additional 132 women who received their care in these clinics were also enrolled for a total of 316 participants.

All participants were pre-menopausal and non-pregnant at the time of entry into the study and were followed at least until they used a course of antifungal therapy for vaginitis, became pregnant or moved from the catchment area. Some patients remained in the study through more than one course of antifungal treatment. No patients asked to prematurely end their participation in the study. The study population had limited options for medical care so that virtually all antibiotic prescriptions were recorded in the records of these patients. Few moved out of the catchment area during the study. None of the participants had vaginal symptoms at the time they entered the study and none had taken antibiotics within 30 days of entering the investigation. Patients in this study were followed for an average of 101 weeks (range, 5–208 weeks).

Patients were followed by repeated review of clinic records, hospital records when appropriate and telephone or personal interviews conducted by the investigator (DDG) or study nurse at intervals of 6 months or less. Data sought included documentation of episodes of antifungal treatment for vulvovaginal candidiasis and confirmed antibiotic treatment or credible history of antibiotic use prior to the use of antifungal therapy. Additional information on these patients was derived from the patient records. We recorded both physician-reported uses of antibiotics and antifungals as well as patient-reported uses of antibiotics and antifungals and requested that patients supply the used medicine packages whenever possible, to increase the reliability of patient-reported histories.

### RESULTS AND DISCUSSION

We followed 316 women for an average of 101 weeks and found that many were treated with antibiotics for a variety of indications during the study. The 316 individuals enrolled used 488 courses of antibiotics while being followed in this investigation. Only 46 individuals (14.5%) reported any use of antifungal therapy for vaginitis and these women used 79 courses of antifungal therapy for an annualized rate of 0.51 presumed cases per year. The majority of antifungal use represented self-treatment (46 uses) and the remainder were prescribed by physician or physician's assistant (33 uses). Some of the patients candidly reported that their use of antifungal therapy was not the result of symptoms but was based on their belief that they would develop a yeast infection after taking an antibiotic.

Those who used antifungal therapy had a history of 97 courses of antibiotics (an average of 2.11 antibiotic uses per patient) whereas the 270 who did not use antifungal drugs reported 391 courses of antibiotics (an average of 1.45 uses per patient) and over all, 29% of women who had a history of antibiotic use also used antifungal therapy, compared to 19% antifungal use among non-antibiotic-treated women (not significant by the Chi-square test). The time of observation was not identical for all patients, therefore when we compared antifungal use on an annualized basis, the rate was 13.8% per year use among antibiotic-treated women versus 11.5% antifungal use per year among those not using antibiotics.

These observations did not distinguish between antibiotic use proximate to the antifungal use versus antibiotic use not temporally associated with the use of antifungal therapy. Other studies[1,4] have considered antibiotic use within approximately 1 month to have relevance to subsequent yeast vaginitis. Therefore we grouped our findings according to antibiotic use within 1 month or 6 months prior to the antifungal use. Table 1 reports antifungal use as the index event and because the antifungal therapy may have represented self-treatment rather than physician-supervised use, we have indicated which cases represent self-treatment. From this information it is clear that only 12% of physician-supervised courses of antifungal therapy were prescribed

**Table 1** Antibiotic use prior to antifungal therapy for yeast vaginitis

| *Interval from antibiotic use to antifungal treatment* | *Courses of antifungal treatment* | *Courses supervised by physician* | *Average time of observation (months)* |
|---|---|---|---|
| 1 month or less | 4* | 4** | 118 |
| 1 to 6 months | 9 | 8 | 110 |
| > 6 months | 41 | 3 | 124 |
| No antibiotic | 25 | 18 | 89 |
| Total | 79 | 33 | 101 |

*One patient self-treated with an antifungal during antibiotic therapy. This patient was not included in this table because we could not state that antibiotic was used prior to antifungal therapy.
**Two patients were treated concurrently with antibiotic treatment by a physician's assistant at the patient's request; also not included in the total within a month of antibiotic use and assuming that antibiotic use greater than 6 months prior to antifungal therapy may be considered as irrelevant to yeast vaginitis, 64% of physician-supervised antifungal therapy events and 84% of all uses of vaginal antifungal treatment were not temporally related to prior antibiotic use.

When all antibiotic use was evaluated, it may be noted that there were four occurrences of antifungal therapy following within a month of antibiotic use, in contrast to 484 antibiotic uses not followed by antifungal use. If the time of observation is extended to 6 months from the index antibiotic use, there were 13 uses of antifungal therapy after antibiotics and 475 uses of antibiotics not followed by antifungal therapy.

A closer examination of the records of the four cases of antifungal use within a month of receiving antibiotic revealed that one patient received an intramuscular injection of ceftriaxone followed by 10 days of oral metronidazole and developed vaginal symptoms 1 day after the metronidazole treatment ended. She did not have an examination to verify the etiology of her vaginal symptoms. A second individual used timethoprim–sulfamethoxazole for 20 days and developed vaginal symptoms 6 days post-therapy. A third patient developed symptoms on the 12th day of a combined regimen of amoxicillin (10 days) and doxycycline (14 days). The fourth individual developed symptoms 29 days after a single 2 g dose of metronidazole. The latter three cases had yeast-positive wet mounts to document the etiology of infection.

This study was not culture-based and did not include a definitive diagnosis of yeast vaginitis. Rather, to attain a larger study population we chose to employ patient use of vaginal antifungal therapy as a surrogate for definitive diagnosis. This would probably overestimate the rate of yeast vaginitis that theoretically would also tend to overestimate any relationship between antibiotic use and yeast vaginitis. While our prospective evaluation of this population could not prove that some cases of yeast vaginitis might be elicited by prior antibiotic treatment, the vast majority of antibiotic uses were not followed by a need for antifungal therapy.

We have previously noted that the concept of antibacterial drugs eliciting yeast vaginitis is commonly repeated in textbooks and ingrained in the thinking of physicians who may vividly recall patients who received an antibiotic prior to developing vaginal symptoms. Furthermore, the concept of a protective role for the vaginal flora is intellectually compelling. However, the literature supporting the concept of antibiotics predisposing to yeast vaginitis is limited to a relatively few reports. Caruso[2] reported that tetracycline treatment was associated with an increase in the percent of women colonized by yeast. A similar result was obtained by Oriel and Waterworth[3] who studied tetracycline and minocyline which provided similar results. Some of the women acquiring yeast in these studies also became symptomatic. For example, in one report, after 14 days of tetracycline the colonization rate increased from 13% to 29% although fewer than half the patients remained in the study. Of the 14 women who were culture positive for yeast, four were reported to have vaginitis[3] and it was also noted that two of the women in the study went from culture positive to culture negative during 2 weeks of tetracycline therapy. They concluded that only a minority of those who had increased yeast colonization actually developed symptoms. One of the four women in our study who used vaginal antifungal therapy after antibiotic had been treated with tetracycline. In contrast, 20 women received tetracycline therapy without developing vaginal symptoms.

The extent to which antibiotics other than tetracycline may affect mucocutaneous yeast is unclear. While our results did not show any consistent increase in antifungal use with antibiotic use, a study by MacDonald and co-workers[4], who also used prescription of antifungal drugs as a surrogate measure of vaginal candidiasis, did show an increase in yeast vaginitis and they also reported that cephalosporins were associated with the greatest attributable risk in these women. Among the 13 women who used antifungal therapy within 6 months of antibiotic treatment only two were treated with a cephalosporin and these were not per oral medication.

The concept that antibiotics, particularly per oral preparations, generally predispose to vaginal yeast infection will probably remain medical dogma, but our results cast doubt on the strength of this association.

### REFERENCES

1. Glover DD, Larsen B. Longitudinal investigation of Candida vaginitis in pregnancy: role of superimposed antibiotic use. *Obstet Gynecol* 1998:91: 115–18
2. Caruso LJ. Vaginal moniliasis after tetracycline therapy. The effects of amphotericin B. *Am J Obstet Gynecol* 1964;90:374–8
3. Oriel JD, Waterworth PM. Effects of minocycline and tetracycline on the vaginal yeast flora. *J Clin Pathol* 1975;28:403–6
4. MacDonald TM, Beardon PH, McGilchrist MM, *et al.* The risks of symptomatic vaginal candidiasis after oral antibiotic therapy. *Q J Med* 1993;86: 419–24

Received 04/30/03; Accepted 07/07/03

## References

[PDF_00319] CARUSO L. J. (1964). VAGINAL MONILIASIS AFTER TETRACYCLINE THERAPY: THE EFFECTS OF AMPHOTERICIN B.. Am J Obstet Gynecol.

[PDF_00315] Glover D. D., Larsen B. (1998). Longitudinal investigation of candida vaginitis in pregnancy: role of superimposed antibiotic use.. Obstet Gynecol.

[PDF_00325] MacDonald T. M., Beardon P. H., McGilchrist M. M., Duncan I. D., McKendrick A. D., McDevitt D. G. (1993). The risks of symptomatic vaginal candidiasis after oral antibiotic therapy.. Q J Med.

[PDF_00322] Oriel J. D., Waterworth P. M. (1975). Effects of minocycline and tetracycline on the vaginal yeast flora.. J Clin Pathol.

